# K_V_1 and K_V_3 Potassium Channels Identified at Presynaptic Terminals of the Corticostriatal Synapses in Rat

**DOI:** 10.1155/2016/8782518

**Published:** 2016-06-09

**Authors:** David Meneses, Ana V. Vega, Francisco Miguel Torres-Cruz, Jaime Barral

**Affiliations:** ^1^Neurociencias (UIICSE), FES Iztacala, Universidad Nacional Autónoma de México (UNAM), 54090 Tlalnepantla, MEX, Mexico; ^2^Carrera de Médico Cirujano (UBIMED), FES Iztacala, Universidad Nacional Autónoma de México (UNAM), 54090 Tlalnepantla, MEX, Mexico

## Abstract

In the last years it has been increasingly clear that K_V_-channel activity modulates neurotransmitter release. The subcellular localization and composition of potassium channels are crucial to understanding its influence on neurotransmitter release. To investigate the role of K_V_ in corticostriatal synapses modulation, we combined extracellular recording of population-spike and pharmacological blockage with specific and nonspecific blockers to identify several families of K_V_ channels. We induced paired-pulse facilitation (PPF) and studied the changes in paired-pulse ratio (PPR) before and after the addition of specific K_V_ blockers to determine whether particular K_V_ subtypes were located pre- or postsynaptically. Initially, the presence of K_V_ channels was tested by exposing brain slices to tetraethylammonium or 4-aminopyridine; in both cases we observed a decrease in PPR that was dose dependent. Further experiments with tityustoxin, margatoxin, hongotoxin, agitoxin, dendrotoxin, and BDS-I toxins all rendered a reduction in PPR. In contrast heteropodatoxin and phrixotoxin had no effect. Our results reveal that corticostriatal presynaptic K_V_ channels have a complex stoichiometry, including heterologous combinations K_V_1.1, K_V_1.2, K_V_1.3, and K_V_1.6 isoforms, as well as K_V_3.4, but not K_V_4 channels. The variety of K_V_ channels offers a wide spectrum of possibilities to regulate neurotransmitter release, providing fine-tuning mechanisms to modulate synaptic strength.

## 1. Introduction

Voltage-dependent potassium channels (K_V_ channels) are crucial for the electrical signaling in neurons. K_V_ channels activate upon depolarization of the plasma membrane, constraining the length of action potentials. Together with calcium-dependent potassium channels (K_Ca_), they are also responsible for the afterhyperpolarization that follows action potentials, thus modulating neuronal firing rates. K_V_ channels are a large family of structurally related proteins with some differences in their biophysical properties, such as voltage range of activation, single channel conductance, kinetics, and behavior of gating [1, 2]. In recent years it has been recognized that different types of K_V_ channels are targeted to different regions within the plasmatic membrane [3–5], but the physiological relevance of this differential sorting is poorly understood. Since K_V_ channels exhibit different sensitivities to kinases and phosphatases, and their activity can be differentially regulated by extra- and intracellular signaling pathways [6–9], it can be predicted that the specific composition of K_V_-channel oligomers will influence local excitability. This is especially important at presynaptic sites where the frequency and shape of action potential are fundamental to determining the timing and strength of synaptic transmission [10].

Short-term forms of plasticity such as paired-pulse facilitation (PPF) are thought to be due to presynaptic modulation, but the mechanisms and molecular targets involved have to be identified precisely [11–13]. Among the molecules involved, potassium channels seem to play a major role [14–16]. In corticostriatal synapses, a role for potassium channels from K_V_ family was first suggested by Jiang and North [14], while studying the modulation of neurotransmitter release by opiates in the corticostriatal synapses. Later, our group showed that blocking K^+^ channels disrupted the opiate-induced downregulation of neurotransmitter release [15, 17]. More recently we have also shown that K_IR_3 channels (also known as GIRK channels) are presynaptically located at corticostriatal synapse and that blocking these channel reduces presynaptic paired-pulse facilitation [18]. In this work we further extend the analysis to investigate the presynaptic expression of K_V_ channels in corticostriatal synapses using the PPF protocol.

When discussing the functional relevance of K_V_ it is important to keep in mind the extraordinary variety of these channels and the complex stoichiometry of its oligomeric structure. K_V_ channels are tetrameric proteins composed of four alpha subunits with six transmembrane segments each that bind together to form the channel pore. Over 40 genes encoding K_V_ alpha subunits have been discovered in mammals, so far. Alpha subunits are organized into 12 families (K_V_1 to K_V_12) with several members each, according to their similarity in sequence, biophysical properties, and pharmacological profiles [6–8]. Alpha subunits from families K_V_1–4, K_V_7, K_V_10, and K_V_11 can combine within their own family to produce functional homo- or heterotetrameric K_V_ channels, while K_V_5-6 and K_V_8-9 families are unable to form functional homomeric channels but can form heteromeric channels with members from K_V_1–K_V_4 families [3, 19]. The reason for such diversity is yet unknown, but it may be necessary to fine-tune the neuronal excitability [2], since the expression of K_V_ channels with different properties can influence the initiation, length, and magnitude of action potentials, both reaching and arising at nerve terminals [4, 10]. Although a high number of combinations are possible, only few combinations have been detected in brain by immunoprecipitation so far (i.e., [20–23]). Here we use a combination of physiological and pharmacological approaches to investigate the composition of K_V_ channels present at the corticostriatal synapse terminals. By using the PPF protocol in combination with selective blockers we set to determine whether a particular isoform of K_V_ is present at the presynaptic site of corticostriatal synapse.

## 2. Material and Methods

Male Wistar rats (100–120 g) provided by the Animal Facilities at FES Iztacala, UNAM, were maintained in accordance with the “Guidelines for the Use of Animals in Neuroscience Research” by the Society for Neuroscience and the Helsinki declaration. Our research center ethical committee approved all protocols.

Sagittal dorsal neostriatal slices (400 *μ*m) were obtained on a vibroslicer (Easislicer, Pelco) and incubated at room temperature in saline solution containing (in mM) 125 NaCl, 3 KCl, 1 MgCl_2_, 2 CaCl_2_, 25 NaHCO_3_, 0.2 (−)-ascorbic acid, 0.2 thiourea, and 11 glucose (saturated with 95% O_2_ and 5% CO_2_, pH: 7.40). Individual slices were transferred to a submerged chamber at 32–34°C. Perfusion rate was adjusted to 1-2 mL/min. Field population spikes, a composite of both field excitatory postsynaptic potential and population action potentials, were recorded with micropipettes filled with 0.9% NaCl (2–4 MΩ) and electrical recordings were obtained using an AC amplifier (P55, Grass Instruments Co., W. Warwick, RI, USA). All recordings were filtered at 1–3 KHz and digitized by PCI-6221 National Instruments (Austin, TX, USA) DAQ (NI-DAQ) board in a PC clone using custom-made programs on LabView*™* environment (National Instruments, Austin, TX, USA). The DAQ board was used to save the data on ASCII or binary files in the computer hard disk for further offline analysis.

Field stimulation was achieved using concentric bipolar electrodes at the cortical white matter. Stimuli consisted of a pair of brief square voltage pulses (4–40 V; 80–200 *μ*s; 0.06–0.4 Hz) generated with an isolated stimulator and delivered through the bipolar electrode. The time interval between the pair of stimuli was in the range of 15 to 50 ms. Bicuculline (10 *μ*M) was added to the perfusion in order to eliminate the inhibitory component of the synaptic response due to GABA_A_ receptor activation [24–26].

A paired-pulse protocol was applied and changes in the responses to paired stimuli were evaluated by calculating the ratio of field-spike synaptic potentials (PPR), which is expressed in percentage as shown in the equation, where *S*_1_ is the first and *S*_2_ is the second orthodromic responses to the stimuli:

(1) $$\begin{array}{c} \%\;PPR=100\times \left(\;\frac{S_{2}}{S_{1}}\;\right). \end{array}$$

Variations in PPR are related to changes in the neurotransmitter release probability in the synaptic terminals from the recorded area [17, 18, 25–28]. This protocol has been widely used to assess changes in neurotransmitter release strength [17, 18, 25, 29–33]. It is generally thought that paired-pulse facilitation (PPF) occurs when the first pulse induces a transient increase of the residual Ca^2+^ concentration in the synaptic terminal, so that the terminal is able to release more neurotransmitter when second pulse arrives, increasing the amplitude of the corresponding field-spike potential (*S*_2_ > *S*_1_). In contrast, paired-pulse depression (PPD) is thought to occur when *S*_1_ is strong enough to deplete the pool of ready-to-release synaptic vesicles. Here, we set the strength of paired pulses to produce PPF and the reduction in the PPR produced by the action of the K^+^ channel blockers was interpreted as a presynaptic effect [15, 17, 18, 25, 34–37]. PPR was measured throughout the experiment and averaged every 5 minutes before and after the addition of drugs.

Blockers for different K_V_ channels were tested at several concentrations. The specificity of K_V_ blockers has been indicated in different studies and reviews; we use the following blockers mainly based in the reviews from Coetzee and coworkers [6] and IUPHAR reviews from Gutman et al. [7, 8]: For K_V_1 channels, dendrotoxin (DTx; Cat. Number: D-350), agitoxin-1 (AgTx; Cat. Number: RTA-150), hongotoxin (HgTx; Cat. Number: RTH-400), margatoxin (MgTx; Cat. Number: STM-325), and tityustoxin (TyTx; Cat. Number: STT-360), for K_V_3 channels, blood depressing substance I (BDS-1; Cat. Number: B-400), and for K_V_4 channels, heteropodatoxin-2 (Cat. Number: STH-340) and phrixotoxin-2 (Cat. Number: STP-710). All toxins were purchased from Alomone (Alomone, Jerusalem). Bicuculline, tetraethylammonium (TEA), and 4-aminopyridine (4-AP) were purchased from Sigma (St. Louis, Mo). The chemicals used in saline solutions were purchased from Sigma-Aldrich or J. T. Baker. They were dissolved from freshly prepared stock solutions into the perfusion saline.

Statistical significance of changes in PPR was assessed with no parametrical tests (Wilcoxon test). The results are expressed as mean ± SEM; other statistical parameters are summarized in Table 1. Dose-response curves were fitted to a Hill equation.

## 3. Results

**Figure 1:**
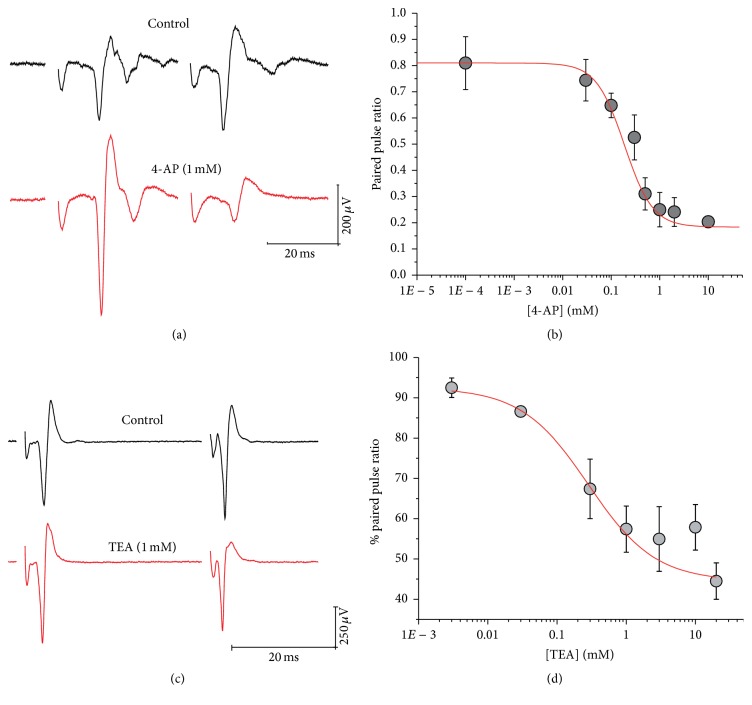
Nonspecific potassium blockers reveal the presence of presynaptic potassium channels at corticostriatal synapses. (a) Exposure to 4-AP (1 mM) causes an increase in the amplitude of the first synaptic response decreasing PPF. Representative traces recorded from the same slice before (upper trace) and after (lower trace) exposure to 4-AP are shown. (c) A similar effect was observed when applying TEA (1 mM). In both cases, paired-pulse facilitation reduction was dependent of the concentration used. (d) and (b), Dose-response relation for TEA and 4-AP, respectively.

We investigate if K_V_ channels may be present at cortical synaptic terminals in striatum, whether these channels were presynaptically located, and how their presence influences synaptic strength. We stimulate cortical areas using a paired-pulse protocol and analyzed the orthodromic response elicited in neostriatum recording field potentials. The population spikes thus elicited were then measured before and after addition of K_V_ blockers 4-AP and TEA (Figure 1).

The corticostriatal synapses were very sensitive to blocking with 4-AP, so that not only was the paired-pulse induced-facilitation abolished, but also paired-pulse depression was induced after the exposure to 4-AP (Figure 1(a)); PPR decreased from 2.63 ± 0.49 to 0.55 ± 0.12 (*n* = 11; *P* < 0.001). Figure 1(a) shows a typical response to 1 mM of 4-AP; the reduction of PPR was close to 130%. The PPR reduction produced by 4-AP was dependent on drug concentration (Figure 1(b)), with an EC_50_ = 0.186 mM. This result indicates the presence of K_V_ channels from families K_V_1 and K_V_3, whose sensitivity to 4-AP falls within the submillimolar rank.

Exposure to TEA at several concentrations also induced a reduction in PPR (Figure 1(c)), although the reduction was not as strong as that observed for 4-AP, even after 20 mM of TEA was applied (see dose-effect curves in Figure 1(d)). In Figure 1(c) we show a representative recording of population spike from a slice before and after exposure to TEA 1 mM. EC_50_ value was calculated to be 0.28 mM. The reduction in % PPR observed with even a little amount of TEA evidenced the presence of TEA-sensitive channels such as K_Ca_1.1 (also known as slo1), the delayed rectifier K_V_1.1, and members of K_V_3 family (type-A channels), as deducted from IC_50_ values reported by others researchers [6, 8].

**Figure 2:**
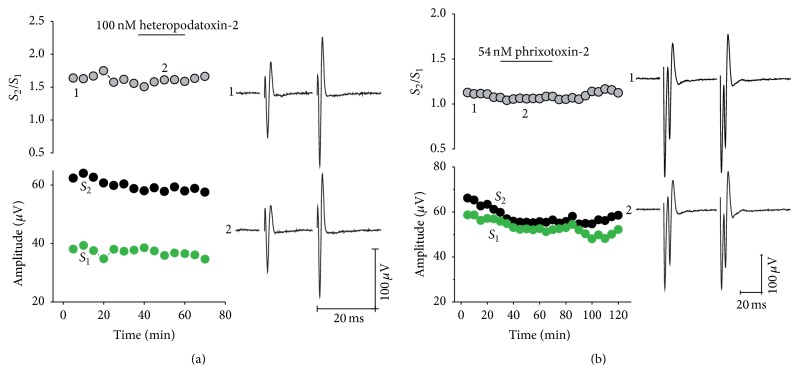
K_V_4 family K^+^ specific blockers do not modify PPF. Representative experiments are shown to exemplify that neither PPR (left upper panels) nor population-spike amplitude (left bottom panels) was modified by exposure to heteropodatoxin (a) or phrixotoxin (b). In this figure and Figures 3 [Fig fig4] [Fig fig5]–6 horizontal bars indicate time of drug perfusion, the traces recorded before (1) and after (2) exposure to K_V_4 toxins are shown on the right panels.

Taken together, these results show that K_V_ channels involved in the release mechanism at the presynaptic terminals are both TEA and 4-AP sensitive, pointing to a K_V_ channel population mainly composed of K_V_1 and K_V_3 channels. For these channels, IC_50_ values for 4-AP blockage or K_D_ values for 4-AP binding below 1 mM have been reported (reviewed by [6]). However, since the TEA-induced reduction in PPR is not as strong as seen for 4-AP, we guess that some K_V_1 isoforms sensitive to TEA in the millimolar range (e.g., K_V_1.2, K_V_1.3, and K_V_1.6) may be also present at corticostriatal nerve terminals. On the other hand, the combination of sensitivities to both drugs led us to think that other families are not present. For example, the A-type K_V_4 channels are insensitive to blockage with TEA and 4-AP, with an IC_50_ within the range of 2–10 mM [6, 8]; therefore it seems quite improbable that K_V_4 channels are represented at corticostriatal synapses. Consistently with this reasoning, the exposure to K_V_4.2- and K_V_4.3-specific blockers heteropodatoxin-2 (100 nM) and phrixotoxin-2 (54 nM) renders no change in PPR (Figure 2). A similar reasoning can be applied to K_V_2, K_V_7, K_V_10, K_V_11, and K_V_12 channels, which are rather TEA and 4-AP insensitive. Within these families IC_50_ for blockage with TEA has been reported to be within 4–28 mM. Likewise, IC_50_ for blockage with 4-AP is calculated to be within 1.5–100 mM [6, 8]. Our results, of course, do not rule out the presence of these channels in other membrane subregions of cortical or striatal neurons or that they play important roles in controlling neuron excitability, but they imply that these channels are not related to neurotransmitter control at corticostriatal presynaptic sites.

**Figure 3:**
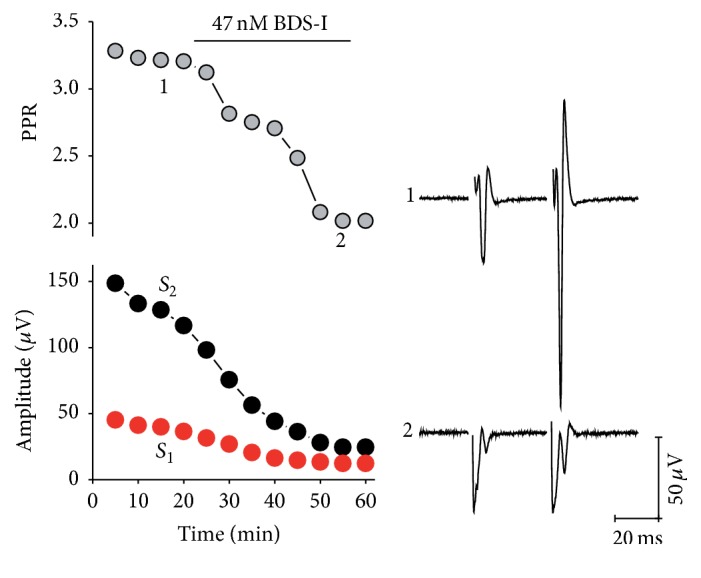
K_V_3.4 channel blockers reduced PPR. The presence of K_V_3.4 channels was revealed blocking corticostriatal synapse with BDS-1 (47 nM). Left superior panel shows time course of PPR of the experiment, and left bottom shows the time course of amplitudes of both *S*_1_ and *S*_2_ synaptic response. Right panel shows representative recordings indicated in left panel.

To confirm the presence of K_V_3 channels we used BDS-I, a K_V_3.4-specific blocker. As shown in Figure 3, exposure to 47 nm of BDS-I results in a strong reduction of PPR from 2.71 ± 0.90 to 1.5 ± 0.40 (*n* = 3; *P* = 0.25). These results strongly suggest the presence of an A-type current playing a major role in presynaptic modulation of the corticostriatal synapses.

**Figure 4 fig4:**
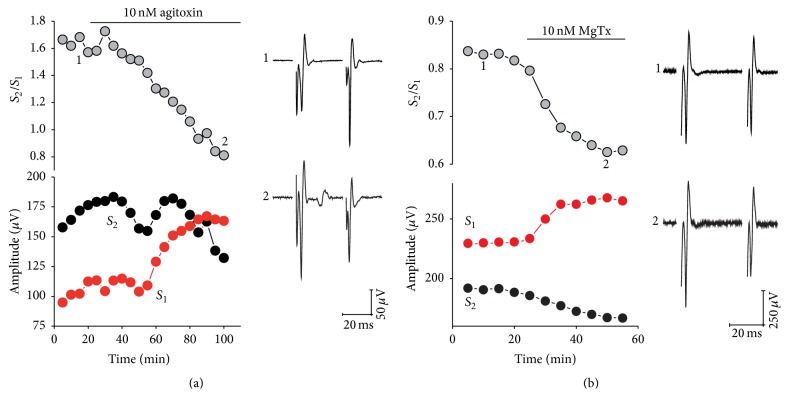
Specific blockers reveal the presence of presynaptic K_V_1 family potassium channels at corticostriatal synapses. Representative experiments showing the reduction of PPR after exposure to (a) agitoxin-1 and (b) margatoxin. In both cases, the upper left panels show the time course of PPR as it changes due to drug administration. Horizontal bars indicate time of drug perfusion. Representative traces showing population spikes before (1) and after (2) exposure to drugs are shown in the right panel. The changes in *S*_1_ (red) or *S*_2_ (black) amplitude are shown on the left lower panels. Initially PPR was adjusted to obtain PPR ≥ 1. Addition of K_V_1 specific blockers results in reduction of PPR that was associated with differential effects on *S*_1_ or *S*_2_ amplitudes.

**Figure 5 fig5:**
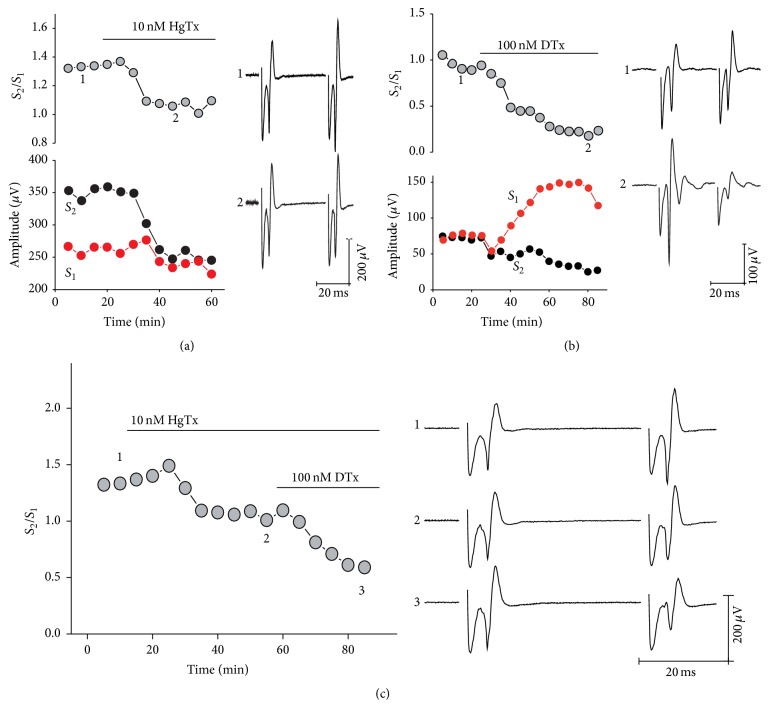
Specific blockers reveal the presence of presynaptic K_V_1.6 family potassium channels at corticostriatal synapses. Representative experiments showing the reduction of PPR after exposure to hongotoxin (a) and dendrotoxin (b) alone or applied sequentially (c). In (a) and (b) upper left panels show the time course of PPR as it changes due to drug administration. Horizontal bars indicate time of drug perfusion. Representative traces showing population spikes before (1) and after (2) exposure to drugs are shown in the right panels. The changes in *S*_1_ (red) or *S*_2_ (black) amplitude are shown on the left lower panels. Initially PPR was adjusted to obtain PPR ≥ 1. In (c), left panel shows the effect on PPR of adding hongotoxin and dendrotoxin sequentially, and traces on the left are shown to exemplify the reduction of PPR.

All members of K_V_1 family are very sensitive to 4-AP; however, sensitivity to blockage by TEA is quite variable. While little amounts of TEA are needed to block homomeric channels K_V_1.1 and K_V_1.6 (IC_50_ 0.5 to 1.7 mM), K_V_1.2 and K_V_1.3 are sensitive to TEA with IC_50_ between 10 and 50 mM, and K_V_1.4, K_V_1.5, and K_V_1.7 are rather insensitive to TEA [6, 38]. However, it has been reported that native potassium channels are found as heteromers rather than homomers and that pharmacological properties in heteromeric channels may differ from homomeric channels, including sensitivity to TEA [38–40]. Therefore, we use several K_V_1 specific blockers alone (Figure 4) or in combination (Figure 5) to explore the presence of several K_V_1 family members.

Although there are no specific drugs for every type of K_V_1 channel, the combination of toxins available gave us a good picture of K_V_1 channels composition. First, we tested agitoxin-1 (10 nM), a specific blocker for K_V_1.3 (Figure 4(a)), and recorded field-potential spikes; once again we observed a reduction on PPR until the point to produce PPD. PPR changed from 1.55 ± 0.05 to 1.01 ± 0.07 (*n* = 6; *P* = 0.008). The effect of agitoxin-1 was established slowly, compared with other toxins tested. This prompts us to think that K_V_1.3 channels might not be expressed as homomeric channels but in combination with other K_V_1 subunits. In support of this idea, when we used margatoxin (10 nM), a K_V_1.2- and K_V_1.3-specific blocker, we observed a reduction in PPR comparable to that observed with agitoxin-1 alone, but the drop on PPR was clearly faster (Figures 4(a) and 4(b)). Margatoxin changed PPR from 1.37 ± 0.20 to 1.10 ± 0.15 (*n* = 6; *P* < 0.05).

**Figure 6:**
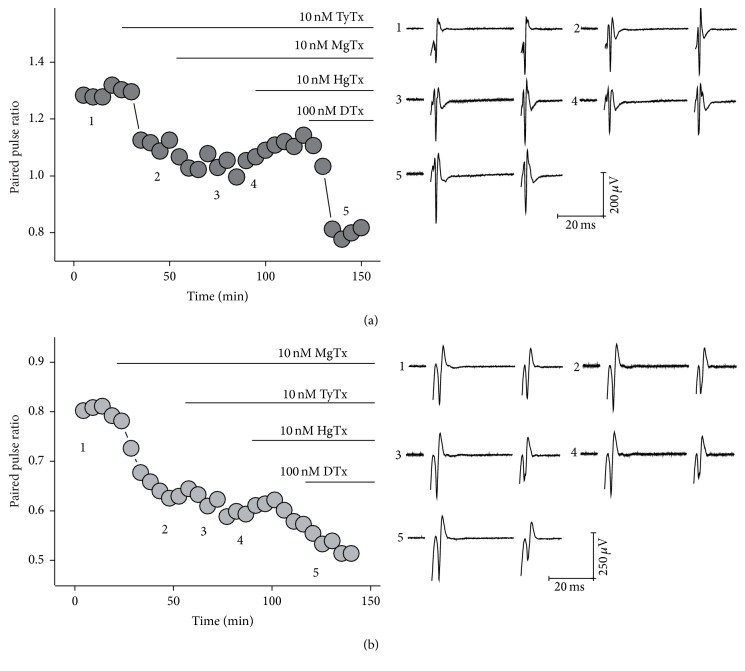
Additive effects of K_V_1 specific-isoforms blockers on PPR. This figure shows the recordings from a single slice sequentially exposed to several K_V_ blockers. An additive effect was observed on PPR (left panels) and traces taken at the times indicated on the PPR plot (right panel). In (a) K_V_1.2 blockage with tityustoxin (TyTx) induces a reduction in PPR that remains with little change despite addition of K_V_1.2/K_V_1.3-blocker margatoxin (MgTX) and K_V_1.1/K_V_1.2/K_V_1.2-blocker hongotoxin (HgTx); however addition of K_V_1.2/K_V_1.3/K_V_1.6-blocker dendrotoxin (DTx) produced a second reduction of PPR. (b) Addition of MgTx to block K_V_1.2/K_V_1.3 channels produced a drop in PPR comparable to that observed with TyTx. Further addition of TyTx and HgTx did not alter PPR. Once again DTx induces a second steep reduction.

In Figures 5 and 6, we show the effects of hongotoxin (10 nM) and dendrotoxin (100 nM) applied alone or in combination. These toxins block equally K_V_1.1 and K_V_1.2 containing channels, but hongotoxin also blocks K_V_1.3 while dendrotoxin blocks K_V_1.6. As expected from the two previous experiments, we observed that hongotoxin renders a significant reduction of PPR, so that PPF becomes PPD; PPR changed from 1.62 ± 0.28 to 1.34 ± 0.10 (17.038%; *n* = 6; *P* < 0.05). In turn, dendrotoxin produced a stronger PPD, 1.30 ± 0.10 to 0.83 ± 0.12 (79.07%, *n* = 14; *P* < 0.001). Consistently with these two results, when HgTx was added sequentially to DTx, it did not render extra reduction of PPR (Figure 6), pointing out the presence of homomeric K_V_1.1 channels at presynaptic sites. In contrast, when dendrotoxin was applied sequentially to hongotoxin (Figure 5) it produced a further reduction of PPR, strongly suggesting the presence of K_V_1.6-containing channels.

In Figure 6 we show the overall effect of K_V_1 channel blockers applied sequentially. When we blocked K_V_1.2-containing channels with tityustoxin (10 nM) we observed PPR reduction, but adding hongotoxin (100 nM) to the bath did not produce further reduction of PPR. This result indicates that K_V_1.2-containing channels are heterotetramers with K_V_1.1 and/or K_V_1.3 and that no K_V_1.1 or K_V_1.3 homotetramers are present at corticostriatal presynaptic terminals. In contrast, addition of DTx (10 nM) renders a second drop in PPR, which points to a K_V_1.6-containing channels population that exists independently of the K_V_1.1/K_V_1.2/K_V_1.3-containing channels population.

Significant PPD was also observed when K_V_1.2/K_V_1.3 channels were blocked with MgTx (10 nM), but little or no further change was observed when applying TyTx (10 nM), as it would be expected if all K_V_1.2-containing channels were already blocked by MgTx. Addition of HgTx (10 nM) did not render further PPR reduction, suggesting again that all K_V_1.2/K_V_1.3 channels were already blocked by margatoxin and tityustoxin. Once again addition of dendrotoxin (100 nM) produced further reduction of PPR, showing the presence of K_V_1.6 channels.

## 4. Discussion

Our results reveal that presynaptic terminals of corticostriatal synapse have several kinds of potassium channel from K_V_ families. Previously, we have shown before that K_IR_ channels also play an important role in modulating neurotransmitter release [18]. Here we showed that K_V_1 and K_V_3 channels are present at these synapses and their blockage with either specific or nonspecific drugs modulates synaptic strength. Our results also rule out the presence of K_V_4 channels at these synapses and imply that presynaptic A-type current can only be carried by K_V_3 channels.

On the other hand, our results indicate that delayed rectifier type currents can be carried by heteromeric K_V_1 channels, resulting from the combination of K_V_1.2, K_V_1.3, and probably K_V_1.1 but also from a population of K_V_1.6-containing channels that are not in combination with K_V_1.1, 1.2, and 1.3. Our observations are in good agreement with previous reports regarding the composition of K_V_ channels. For example, Koch et al. [40] reported that all ^125^I-margatoxin receptors contain at least one K_V_1.2 subunit and over 80% also contain K_V_1.1; additionally ~30% of these receptors contain K_V_1.3 subunits. Specific inhibitors for K_V_1.3 and K_V_1.2 are able to reduce PPR, but kinetic and strength of blocking are remarkably different. Specifically K_V_1.3 inhibitor agitoxin-1 seems to be less effective than K_V_1.2 inhibitor tityustoxin, which could be related to the relative abundance of both subunits. We observed that hongotoxin does not increase PPR reduction after K_V_1.2/K_V_1.3 channels have been already blocked with tityustoxin and margatoxin (Figure 6); therefore if K_V_1.1 is present at presynaptic action site, it should be associated with K_V_1.2 and/or K_V_1.3 rather than being homomeric. In contrast, the addition of dendrotoxin after K_V_1.1/K_V_1.2/K_V_1.3-containing channels had been blocked with hongotoxin renders an extra reduction of PPR, pointing toward an independent K_V_1.6-containing channel population, with a composition that excludes K_V_1.1, K_V_1.2, and K_V_1.3. It has been proposed before [6, 41] that the combination of K_V_1.1/K_V_1.2/K_V_1.6 accounts for a slow IA, named D-type current, that is sensitive to low concentrations of dendrotoxin and 4-AP; the existence and role of such currents in regulating synaptic release surely deserve further investigation.

In cortical neurons it has been observed that K_V_-channel alpha subunits are differentially distributed among dendrites, soma, axon, and axonic terminals. For example, somatic action potentials are sensitive to both TEA and 4-AP, but axonic action potential is only sensitive to 4-AP, indicating a different composition of heteromeric K_V_1 channels. Moreover, pharmacological blockage of K_V_ channels with dendrotoxin-I increases the synaptic latency, probably due to a shift of the presynaptic calcium current resulting from the prolonged axonal spike [10]. However, the repertoire of channels at presynaptic boutons should also be taken into account since action potential at presynapsis strongly modifies the action potential width and, consequently, neurotransmitter release [42].

Expression of K_V_ channels in pyramidal cells has been investigated more extensively in hippocampus. All K_V_1 channels have been detected by immunocytochemistry in hippocampal stratum pyramidale from mouse, but only K_V_1.1, K_V_1.2, and K_V_1.6 are located in the stratum oriens, while the K_V_1.3 immunoreactivity is absent in this layer [43]. An earlier report in rat hippocampus showed that K_V_1.1 was prominent in stratum lucidum of CA3, while immunostaining for K_V_1.2, K_V_1.3, and K_V_1.6 was very weak and it was difficult to determine whether the signal belongs to the dendritic area of pyramidal CA3 neurons or the mossy fiber terminals [44]. Interestingly, K_V_1.1 immunoreactivity in stratum lucidum disappears when dentate gyrus neurons are exposed to excitotoxic agents [45], which means that K_V_1.1 is located in mossy fibers terminals rather than CA3 dendrites. In contrast, kainic acid lesions of CA3 strongly reduced K_V_1.1 immunoreactivity in stratum radiatum, indicating that K_V_1.1 is associated with axons and terminal of Schaffer collaterals [45]. In good agreement with these observations, a recent report shows that K_V_1.1 is present on axon initial segments (AIS) and axon terminals by freeze-fracture gold labelling and that density is 8 times higher on AIS [46]. In contrast with the report from Veh et al. [44], Sheng and coworkers [47] have reported that K_V_1.2 immunoreactivity is concentrated on dendrites in hippocampal and cortical pyramidal cells [47, 48] and is conspicuously absent in cell bodies. Interestingly, excitotoxic lesion of entorhinal cortex reduced the density of K_V_1.2 in stratum moleculare of CA1–CA3. Our results are in good agreement with these observations, as we have shown that K_V_1.1 and K_V_1.2 are present at cortical axon terminals. Presynaptic localization of heteromeric K_V_1.1/K_V_1.2 channels has been observed in primary cultures [49]; likewise K_V_1.3 is thought to be targeted preferentially to the axon [50]; our results provide functional evidence that K_V_1.1/K_V_1.2/K_V_1.3 complexes are located presynaptically in vivo and that they modulate neurotransmitter release. Interestingly, in layers II/III from somatosensorial and motor cortex mRNA for K_V_1.1 to 1.6 can be detected, but immunoreactivity for K_V_1.6 seems to be almost absent within the cortex [46]. A possibility is that K_V_1.6 is sorted to axon terminals, accounting for a dendrotoxin-sensitive population of K_V_1.6 channels located at corticostriatal synapses.

Regarding K_V_4 we did not find evidence of the presence of these channels at presynaptic terminals; this is in agreement with previous studies showing that K_V_4 channels are selectively transported to dendrites in cortical neurons in culture [51]. Instead we found evidence for the presence of K_V_3.4 channels, which are both TEA and 4-AP sensitive. In hippocampal pyramidal cells 87% of cells express mRNA for K_V_4 channels, while only 17% express mRNA for K_V_3 (K_V_3.1 and K_V_3.2) [52]; however Jinno and coworkers [53] have shown that K_V_4.2 is located in somatic and dendritic compartment, which is in good agreement with our observations.

In support of the interpretation of our experiments regarding the pre- or postsynaptic localization of K_V_s, it is worth mentioning that in striatal medium spiny neurons mRNA for K_V_1.3 is absent, but K_V_1.2 is present. In these neurons K_V_1.2 seems to account for as much as 50% of subthreshold depolarization-activated K^+^ current. Interestingly, margatoxin fails to block this somatic current [41]. In contrast, in our experiments margatoxin has a strong effect, supporting the notion that the K_V_1.2/K_V_1.3-containing channels are concentrated on the presynaptic side of corticostriatal synapses.

K_V_1 channels have been reported to be presynaptically located in other glutamatergic synapses [9]. For example, mossy fibers from dentate granule cells show prominent immunolabeling for K_V_1.1 and K_V_1.4 in preterminal segments [21, 54]. Accordingly, fast inactivating dendrotoxin-sensitive TEA-insensitivity currents are recorded in outside-out patches isolated from mossy fibers [47]. However, while it has been suggested that K_V_1.6 is not present in mossy fibers boutons, in corticostriatal synaptic terminals we found evidence that there is an important population of K_V_1.6 channels. On the other hand, K_V_3 channels have been observed to be located presynaptically in cerebellar stellate interneurons and sympathetic nerves [42, 45]. In interneurons from cerebellar molecular layer K_V_3 channels are segregated from K_V_1 channels, located in soma and the most proximal axon region, while K_V_3 channels are located in presynaptically buttons [42].

There is a growing body of evidence highlighting that K^+^ conductances located at presynaptic terminals control the release of transmitter [3, 12, 18, 55–57]. For example, it has been reported that DTX-sensitive K^+^ currents are modulated by opioid signaling and play an important role regulating the calcium influx into mossy fibers terminals [45, 58]. In corticostriatal synapses, glutamatergic release is also regulated by opiates and regulation of K^+^-channels activity is part of the modulation mechanism fibers [14]. *μ*- and *δ*-opiate receptors facilitate glutamate release from corticostriatal [15, 17]. Since blocking potassium channels with 4-AP and Ba^2+^, but not TEA (1 mM), precludes opiate upregulation of glutamate release, it was suggested before that K_V_4 may be involved in the neuromodulation by opiates [15]. However, our study shows that K_V_4 specific blockers do not modify PPR, indicating that K_V_4 channels are not expressed presynaptically. As it has observed in mossy fibers [21, 54], heteromeric K_V_1.1-containing channels may be the ones involved in opiate-induced PPF. A K_V_1.1 heterotetramer containing K_V_1.2 and/or K_V_1.3 would fit the pharmacological profile of potassium channels involved in opiate-induced modulation, sensitive to 4-AP and Ba^+2^ and relatively insensitive to TEA, since the presence of K_V_1.2 in K_V_1.1/K_V_1.2 homotetramers renders an IC_50_ for TEA of 10 mM [39].

Neurotransmitter release from vesicles lasts only a few milliseconds, due in part to the balance between the activation of voltage-gated calcium and potassium currents. Upregulation of K_V_-channels activity presumably will lead to a stronger and faster repolarization, reducing the time window for calcium influx trough voltage-dependent Ca^2+^ channels, with the consequent reduction in the neurotransmitter release. The present study shows that K_V_ conductances effectively are present at presynaptic terminals of corticostriatal afferents and modulate glutamate release at these synapses. In a previous report we showed that these terminals are also endowed with K_IR_3 channels [18], which can be regulated by several G-protein receptors. Altogether our results show that corticostriatal synaptic terminals are endowed with multiple types of voltage-gated potassium channels, and this opens a large pool of targets/tools for neuromodulators to regulate the synaptic action potential achieving thereby synaptic plasticity.

## Figures and Tables

**Table 1:** Effects of K_V_ channels blockers on PPR. Statistical summary.

	Before			After			% PPR	*P*	*n*
	PPR	Median	Range	PPR	Median	Range			
TEA	2.35 ± 0.58	1.96	1.21–5.49	1.35 ± 0.29	0.99	0.84–3.02	60.98	*∗∗*	7
4-AP	2.63 ± 0.49	1.97	1.08–5.56	0.55 ± 0.12	0.36	0.07–1.11	76.93	*∗∗*	11
Heteropodatoxin	1.31 ± 0.33	1.31	0.98–1.64	1.26 ± 0.28	1.26	0.98–0.15	25.68	NS	3
Phrixotoxin-2	1.09 ± 0.12	1.12	0.87–1.30	1.28 ± 0.12	1.28	1.07–1.45	17.01	NS	4
BDS-1	2.71 ± 0.90	3.21	0.97–3.97	1.57 ± 0.40	1.61	0.85–2.24	33.90	NS	3
Agitoxin-1	1.55 ± 0.05	1.55	1.42–1.68	1.01 ± 0.07	1.01	0.81–1.20	19.70	*∗*	6
Margatoxin	1.37 ± 0.20	1.44	0.76–2.04	1.10 ± 0.15	1.13	0.62–1.62	20.87	*∗*	6
Hongotoxin	1.62 ± 0.28	1.42	1.14–2.69	1.34 ± 0.23	1.06	1.00–2.36	17.04	*∗*	6
Tityustoxin	1.37 ± 0.21	1.44	0.59–1.72	1.05 ± 0.16	1.02	0.59–1.72	18.49	NS	6
Dendrotoxin	1.30 ± 0.10	1.20	0.85–2.15	0.83 ± 0.12	0.73	0.21–1.74	79.07	*∗∗*	14

^*∗∗*^ *P* < 0.001;  ^*∗*^ *P* < 0.05; NS: no significance.
